# Vitamin D and Human Health

**DOI:** 10.3390/ijms20010145

**Published:** 2019-01-03

**Authors:** Michal A. Zmijewski

**Affiliations:** Department of Histology, Medical University of Gdańsk, 80-211 Gdańsk, Poland; mzmijewski@gumed.edu.pl

**Keywords:** vitamin D, analogs of vitamin D, vitamin D deficiency, supplementation, vitamin D activity and metabolism, extra-skeletal effects of vitamin D, therapy and prevention

## Abstract

Vitamin D is currently one of the hottest topics in research and clinics, as well as in everyday life. Over the past decades, scientists gathered overwhelming evidence indicating that the observed global vitamin D deficiency not only has a negative impact on human skeletal system, but also facilitates development and progression of multiple disease of civilization, including cardiovascular diseases, diabetes, autoimmune disease, and cancer. This Special Issue, entitled “Vitamin D and Human Health”, summarizes recent advances in our understanding of pleiotropic activity of vitamin D in the form of eight comprehensive reviews. Furthermore, eight research papers provide new insight into vitamin D research and highlight new directions.

## 1. Introduction

The active form of vitamin D (1,25(OH)2D3, calcitriol) regulates calcium–phosphate homeostasis through the interaction with vitamin D receptor (VDR). It also has a huge impact on the proper functioning of musculoskeletal, immune, nervous, and cardiovascular systems. It is well known that despite huge progress, the technical revolution caused substantial changes in the environment and a human life. An introduction of diets based on highly processed food, an indoor lifestyle, and sun avoidance greatly contributed to the development of the global vitamin D deficiency. A low level of vitamin D is strongly correlated with a decreased calcium level, which in turn leads to inadequate mineralization of bones with subsequent development of rickets in children or osteoporosis in adults. It results not only in bone deformation, but also in high susceptibility of falls and bone fractures. Thus, proper vitamin D supplementation according to recent standards is essential for maintenance of the body homeostasis [1,2,3,4]. In spite of tremendous efforts and accumulating data concerning the impact of vitamin D on human life, there is still the need for extensive studies on molecular mechanisms activated by vitamin D, which would underline potential benefits of this pleiotropic hormone. On the other hand, clinical significance of vitamin D needs to verified through a series of large, randomized, controlled long-term trials based on comparison of serum levels of 25(OH)D3 rather than doses of supplementations.

## 2. Vitamin D and Health

Vitamin D deficiency is inseparably connected to demineralization of bones, which results in an increased susceptibility to fractures. Atteritano and coworkers presented a case study showing the relation between low vitamin D levels and susceptibility to bone fragility fractures in HIV-positive patients [5]. De Luca et al. showed that the presence of specific alleles of *Fok*I single nucleotide polymorphism (SNP) in the vitamin D receptor (*VDR*) gene affects cell proliferation and inflammatory response. The study was contacted on intervertebral disc cells derived from patients with discopathy or spondylolisthesis. Interestingly, it seems that the *Ff* variant of *Fok*I genotype of the *VDR* gene is more responsive to the anti-inflammatory effects of vitamin D and could be used as a diagnostic factor in discopathies [6]. Furthermore, the effects of the *VDR* SNPs were also investigated in acute pancreatitis (AP). It was found that a particular SNP (allele T in *Taq*1) is almost three times more frequent in AP patents in comparison with alcohol-abuse controls [7]. Thus, it seems that alteration in vitamin D signaling through the presence of unique SNPs in the *VDR* gene may be associated with predisposition to certain pathologies.

The involvement of vitamin D in the regulation of the functions of the cardiovascular system and its pleasurable impact on hypertension is currently under debate and intensive investigation. Legarts et al. [8]. summarized our current knowledge concerning the role of vitamin D in regulation of blood pressure and development of hypertension. It has to be underlined that multiple animal studies involving VDR-null mutants have shown that vitamin D has a direct impact on regulation of the renin–angiotensin–aldosterone axis and *VDR* mutations, or a low level of vitamin D results in an increase in the blood pressure. However, human trials or retrospective studies have not found a clear association between vitamin D level or its supplementation with hypertension. It could have been the problem with suboptimal study design and hopefully ongoing large scale, randomized studies will validate potential benefits of vitamin D in the treatment of hypertension.

Vitamin D deficiency is often association with several neurological diseases, as the vitamin D receptor is expressed in several brain structures including the hippocampus, hypothalamus, substantia nigra, and thalamus. Most importantly, vitamin D regulates the expression of neurotrophins, including neural growth factor (NGF) and neurotransmitters (acetylcholine, dopamine, and gamma-aminobutyric acid). In the current issue of *IJMS*, Morretti and coworkers [9] summarized our current knowledge concerning the role of vitamin D in the prevention and treatment of neurological disorders, focusing on multiple sclerosis, stroke, and Alzheimer’s and Parkinson’s diseases.

## 3. Vitamin D and Immune Response

It is well established that vitamin D inhibits proliferation and induces differentiation of the cells of different linages and is essential for regeneration of the epithelial barrier, as well as maturation of immune cells. For example, lymphocytes, neutrophils, monocytes, and dendritic cells not only express VDR and are direct targets for 1,25(OH)2D3, but also activate circulating 25(OH)D3 through hydroxylation by CYP27B1 [10]. The immunomodulatory effects of 1,25(OH)2D3 include switching between cell-mediated response (Th1) and humoral immunity (Th2). Vitamin D activates macrophages and production of antimicrobial peptides by epithelial and immune cells, which could be essential in the eradication of bacterial or viral infections. It is not surprising that an occurrence of the sessional infections, such as influenza, is often linked to vitamin D deficiency. Keeping in mind the various effects of vitamin D on immune response, Gruber-Bzura [11] discussed the potential role of vitamin D in influenza prevention and treatment. It has to be underlined that an impact of vitamin D on the immune system is usually cell type, tissue, or organ dependent. For instance, it was recently suggested that vitamin D could be useful in the prevention and treatment of autoimmune diseases such as multiple sclerosis, type 1 diabetes mellitus, rheumatoid arthritis, or systemic lupus erythematous (SLR). The consequence of vitamin D deficiency in the lupus development and progression was reviewed by Mak [12]. Strikingly, exposure to UV light is a major contributor to SLR flare up, thus the sun avoidance behavior only aggravates vitamin D deficiency in patients with lupus. On the other hand, a few recent clinical studies suggested not only a correlation of vitamin D deficiency with the severity of lupus, but also that proper supplementation may inhibit the production of autoantibodies, decrease the Th1/Th17 and memory B cells fractions, and reduce fatigue [12]. Furthermore, an increased activity of the immune system, including production of specific antibodies, is also the most important cause of graft-versus-host disease in recipients of allogeneic hematopoietic stem cell transplantation. Thus, the modulatory role of vitamin D may decrease adverse effects of graft-versus-host disease [10].

## 4. Vitamin D and Cancer 

It is well established that the low level of vitamin D is associated with an increased risk of any type of cancer and a decrease survival rate, mainly because of an increased severity of the symptoms and metastatic potential of malignancies [13]. Very promising clinical studies analyzed by Medrano [10] suggested that vitamin D supplementation is significantly associated with an increase in overall survival and lower risk of relapse of myeloid, but not lymphoid malignancies in transplant recipients. The possible link between vitamin D and an immune regulation of the tumor microenvironment was also discussed by Liu et al. [13]. It is well established that vitamin D modulates an immune response through the inactivation with the NFκB pathway. In the tumor stroma, secretion of cytokines and prostaglandins is essential for the propagation of cancer cells, but vitamin D, through the downregulation of NFκB and cyclooxygenase 2 (COX-2), can attenuate their secretion. On the other hand, Pawlik and coworkers [14] observed that vitamin D and its analogs (PRI-2191 and PRI-2205) modulate the prevalence of a certain fraction of lymphocytes (an increase number of T helper lymphocytes (Th2), regulatory T (Treg), granulocytes, and B lymphocytes), but reduce the fraction of TCD4+, TCD4+CD25+, and TCD8+ cells in the 4T1 mouse mammary gland cancer model. It was accompanied by the modulation of the level of pro-tumorogenic cytokines in the serum. It seems that the modulatory effects of vitamin D in a cancer treatment may also include the adverse effects, which should be considered.

Cancer metastasis is the most important problem in the treatment of any type of cancer. For instance, in melanoma, metastasis dramatically decreases the survival rate of patients [15]. Many studies have shown recently that vitamin D and its analogs can be used in adjuvant radio-therapy (see recent review [16]). In the current issue of *IJMS*, Podgórska et al. [17] documented that treatment with either 1,25(OH)2D3 or 25(OH)D3 sensitized human (SKMEL-188) and Bomirski’s hamster melanoma cells to low doses of proton beam radiation. Interestingly, vitamin D is also considered in the treatment of benign tumors such as uterine fibroids, derived from smooth muscle cells of the uterus. As reviewed by Ciebiera and coworkers [18], a few clinical studies have shown that low serum levels of 25(OH)D3 or the presence of specific SNPs of the genes related to vitamin D metabolism or activity correlate with the occurrence of uterine fibroids. Thus, keeping in mind antiproliferative and antifibrotic properties of vitamin D, authors suggested its potential beneficial effects not only in prevention, but also in the treatment of uterine fibroids [18].

## 5. Vitamin D Analogs

For many years, both supplementation and clinical uses of vitamin D were limited because of the potential occurrence of hypercalcemia. Thus, many laboratories around the world have investigated vitamin D analogs, which do not affect calcium level, but still possess antiproliferative and immunomodulatory properties of the active form of vitamin D. Hundreds of synthetic analogs have been investigated so far, with some pleasurable effects. Interestingly, resent studies have shown that not only does 1,25(OH)2D3 possesses biological activity, but also its precursor, 25(OH)D3, could effectively inhibit proliferation of melanoma cells [19] or be used as a radio-sensitizing agent in the melanoma treatment [17]. It is also well established that the cholesterol side-chain cleavage enzyme P450scc (*CYP11A1*) could catalase the synthesis of several vitamin D hydroxyderivatives. Those compounds were shown to be the potent inhibitors of a cell proliferation with immunomodulatory properties (see recent review [15]). Slominski and coworkers [20] have demonstrated that one of the products of CYP450ssc enzymatic activity, 20,23(OH)2D3, and the active form of vitamin D (1,25(OH)2D3) share similar, but also activate unique genomic targets. This observation could at least partially explain the decreased impact of 20,23(OH)2D3 on the serum level of calcium in comparison with calcitriol. In the current issue of *IJMS*, Wasiewicz et al. [19] compared the antiproliferative activity of 1,25(OH)2D3, synthetic calcipotriol, and a short side-chain vitamin D analog 21-hydoxypregnacalciferol (21(OH)pD) on three melanoma cell lines. Interestingly, it was shown that the antiproliferative activity of 21(OH)pD was not fullly dependent on the expression of VDR. This particular observation could be of great importance because in melanoma (like in many other cancers), a decreased level of VDR correlates with disease progression [15]. Finally, diverse effects of two vitamin D analogs, (24*R*)-1,24-dihydroxyvitamin D3 (PRI-2191) and 5,6-*trans* isomer of calcipotriol (PRI-2205), on the tumor microenvironment and metastasis of 4T1 mouse mammary gland cancer were studied by Prof. Wietrzyk’s group ([14] and see discussion above).

## 6. New Cellular Targets for Vitamin D and Its Analogs

It is well established that the active form of vitamin D (1,25(OH)2D3) binding to the VDR–RXR complex and its subsequent translocation to the nucleus activates the classic genomic pathway. However, the existence of a fast nongenomic vitamin D response with the involvement of cell membrane bond VDR and/or protein disulfide isomerase PDIA3 was also postulated [2]. The modulation of immune response by nongenomic pathway was also discussed by Medrano [10]. Interestingly, a recent study from Prof Slominski’s group [20] suggested that an aryl hydrocarbon receptor (AhR) is a new unique target for 20,23(OH)2D3. This unexpected observation has opened new therapeutic options for this unique vitamin D analog.

Recently, mitochondria have been recognized as a potential target for the action of vitamin D. Ricca and colleagues [21] showed that VDR plays a crucial role in the regulation of mitochondrial respiration and protects cells from an excessive production of reactive oxygen species (ROS) and subsequent cell damage. This is in line with our recent observation that vitamin D and its analogs modulate mitochondrial membrane potential; production of reactive oxygen species (ROS); and expression of ROS-associated genes, including catalase and superoxide dismutases (SOD1 and SOD2) [22]. On the other hand, Abu el Maaty and coworkers have recently investigated the potential targeting of thioredoxin-interacting protein (TXNIP) by vitamin D [23]. TXNIP is known to play a pivotal role in the regulation of glucose and redox homeostasis and its expression was shown to be modulated by vitamin D. However, the current study [23] postulated more complex interactions between vitamin D and TXNIP. The effects of vitamin D on TXNIP expression were shown to be cancer cell line specific and glucose dependent. Furthermore, there are also indications that vitamin D affects TXNIP protein stability during prolongated incubation.

Finally, our recent studies showed that VDR is not fully required for antiproliferative activities of short side chained analogs of vitamin D such as 21-hydroxypregnacaliferol (21(OH) pD [19,24]. However, the potential intracellular pathways activated by these vitamin D analogs still remain to be discovered.

## 7. Conclusions

This Special Issue gives insight into the evolving field of vitamin D regarding its mechanisms of action, deficiency, supplementation, health benefits, and clinical applications.

There is ongoing debate as to whether vitamin D should be treated only as a supplement, eventually used in prophylactics, or if it could be also considered in the therapy of multiple disorders. Having in mind pleiotropic, modulatory effects of vitamin D, the serum level of 25-OH D3 should be always considered as an important diagnostic factor, especially in the case of vitamin D deficiency. Multiple clinical trials also showed positive effects of vitamin D supplementation on overall human health, and suggested its possible use in the treatment of several diseases, including cancer. However, further large studies are still required in order to validate the potential benefits and safety of vitamin D in clinics. On the other hand, low calcemic analogs are a very promising alternative for calcitriol, and new pathways activated by vitamin D and its analogs broadened our knowledge concerning the role of vitamin D in human health and disease.
